# Liquid Chromatography–High-Resolution Mass Spectrometry (LC-HRMS) Profiling of Commercial Enocianina and Evaluation of Their Antioxidant and Anti-Inflammatory Activity

**DOI:** 10.3390/antiox11061187

**Published:** 2022-06-16

**Authors:** Larissa Della Vedova, Giulio Ferrario, Francesca Gado, Alessandra Altomare, Marina Carini, Paolo Morazzoni, Giancarlo Aldini, Giovanna Baron

**Affiliations:** 1 Department of Pharmaceutical Sciences (DISFARM), Università degli Studi di Milano, Via Mangiagalli 25, 20133 Milan, Italy; larissa.dellavedova@unimi.it (L.D.V.); giulio.ferrario1@unimi.it (G.F.); francesca.gado@unimi.it (F.G.); alessandra.altomare@unimi.it (A.A.); marina.carini@unimi.it (M.C.); giancarlo.aldini@unimi.it (G.A.); 2 Divisione Nutraceutica, Distillerie Umberto Bonollo S.p.A, Via G. Galilei 6, 35035 Mestrino, Italy; paolo.morazzoni@bonollo.it

**Keywords:** enocianina, enocyanin, grape pomace, waste, anthocyanins, catechol, Nrf2, antioxidant, anti-inflammatory

## Abstract

Enocianina is an anthocyanin-rich extract obtained from grape pomace. It is widely used as a colorant in the food industry and, in addition to anthocyanins, it also contains a variety of polyphenols. To understand whether enocianina, besides its coloring effect, may offer potential health benefit applications, we aimed to fully characterize the profile of four commercial enocianinas and assess their radical scavenging, enzymatic, antioxidant, and anti-inflammatory activities. LC-ESI-MS/MS analysis identified 90 phytochemicals. The relative content of each anthocyanin was assessed by a semi-quantitative analysis, with malvidin derivatives being the most abundant. UV-VIS spectroscopy detected total amounts of polyphenols and anthocyanins of 23% and 3.24%, respectively, indicating that anthocyanins represent a minor fraction of total polyphenols. Multiple linear regression analysis indicated that the radical scavenging activity is related to the total polyphenol content and not to anthocyanins. All four enocianinas dose-dependently activate Nrf2, and such activity was correlated with catechol-containing polyphenol content. Finally, all enocianinas showed dose-dependent anti-inflammatory activity, which at the highest concentrations tested was closely related to the total polyphenol content and was explained by radical scavenging, Nrf2 activation, and other mechanisms related to the polyphenolic components.

## 1. Introduction

Anthocyanins (ACNs) are a class of more than 700 naturally occurring pigments (orange, red, violet, and blue) extensively distributed in nature, and are members of the flavonoid class [1]. Several anthocyanin-rich extracts, isolated from fruits and vegetables, including grape skin, currant, elderberry, cranberry, bilberry, maize, cabbage, and carrot, are widely used as food colorants [2]. Oenocyanin, enocyanin, or enocianina is the name of anthocyanins when extracted from grape pomace, composed of the stalks, skin, pulp, and seeds that are left over after grape pressing during the winemaking process [3,4]. Grape pomace is available in large amounts, and this poses ecological and economic difficulties. Wineries often use these by-products as fertilizers or animal feed, and sometimes sell them to biogas plants to produce renewable energy [5]. However, wine production waste has a much higher potential, given the amount of valuable chemical compounds that can be recovered from it [6]. Part of grape pomace is used to produce enocianina, which is widely used for the food industry (E-163, grape peel extract) and, in particular, for the pigmentation of drinks, liqueurs, yogurt, ice creams, etc. [4].

From a historical perspective, Antonio Carpenè and Enrico Comboni first reported and applied the industrial production of enocianina from grape pomace in 1879 [7]. Hence, enocianina could be considered as an early example of circular economy, where a waste product of the wine industry (grape pomace) is recycled to generate useful materials (food colorants).

Different methods for enocianina production have since been reported, aimed at removing some or all of the saccharides/polysaccharides whilst retaining the anthocyanins containing polyphenol fraction. Traditionally, pomace from deeply pigmented grape cultivars such as Lancellotta, Lambrusco, Alicante, and Salamina is extracted batch-wise with decreasing concentrations of sulfur dioxide [8]. Sulfur dioxide facilitates the solubilization of anthocyanins due to the transient formation of more water-soluble complexes. After concentration, the liquor may be refrigerated to remove potassium tartrate, centrifuged, and formulated as a liquid or a spray-dried solid. Others have experimented with different extraction solvents [9,10], supercritical carbon dioxide [11], and, more recently, eutectic solvents [12,13].

Enocianina is usually characterized as the total content of anthocyanins (colorimetric method), and relative/absolute content of the most abundant anthocyanin components deriving from the five anthocyanosides (cyanidin, delphinidin, malvidin, peonidin, and petunidin) has been reported using commercial and lab-scale enocianina preparations, with malvidin-3-O-glucoside and peonidin-3-O-glucoside being the most abundant [12,14]. Besides anthocyanins, enocianinas contain other polyphenolic bioactive compounds, belonging to the original material, particularly flavonols, which are found in the hypodermis, the inner layer of the grape skin [4]. Other constituents are represented by sugars, organic acids, and inorganic materials. 

Despite its wide application, it should be noted that a detailed qualitative profile of enocianina, including minor components, has not yet been obtained. This aspect has been underlined by the EFSA Panel on Food Additives and Nutrient Sources added to Food (ANS), which has recommended a full qualitative and quantitative profiling of enocianina to permit an adequate risk evaluation for derivation of an Acceptable Daily Intake (ADI) for anthocyanin (E 163) as food additives [15].

Enocianina, besides being used as coloring agent, also represents a source of bioactive compounds (anthocyanins and other polyphenolic compounds) with many pharmacological effects: antioxidant, anti-inflammatory, and the prevention of age-related chronic diseases, such as cardiovascular disease (CVD), cancers, neurodegenerative, and eye-related diseases [16,17]. Regarding the molecular mechanisms, the anti-inflammatory activity of anthocyanins containing a catechol moiety, such as delphinidin and cyanidin derivatives, has been mainly attributed to the Nrf2 pathway, which also explains the in vivo antioxidant activity [18].

Anthocyanins also have antiviral properties. Recent in vitro studies have shown that they can inhibit the replication of viruses such as herpes simplex, parainfluenza virus, syncytial virus, HIV, rotavirus, and adenovirus [16,19]. The broad spectrum of pharmacological properties supported by preclinical and clinical evidence, associated with a low toxicity, make their pharmacotherapeutic use very attractive.

Besides anthocyanins, enocianina also contains other flavonoidic classes including flavonols, flavanols, stilbenoids, catechins, and phenolic acids, which also possess antioxidant, anti-inflammatory, and antimicrobial properties [4]. 

Taking into account that enocianina is a valuable and commercially available source of bioactive compounds, that it is a safe extract with a long traditional use, and which benefits the circular economy, the aim of the present paper is to better understand whether this extract can be used, in addition to its food coloring effect, also for its potential health benefits. 

To reach this goal, the first step is (1) to fully characterize, by the LC-HRMS approach, in positive and negative ion modes, the qualitative profile of enocianina; (2) to establish the semi-quantitative profile of each anthocyanin; (3) to assess the total anthocyanin, tannin, and polyphenols content; (4) to evaluate the antioxidant and anti-inflammatory activities. Analyses were carried out on four commercial enocianinas to evaluate their heterogeneity in terms of composition and activity. 

## 2. Material and Methods

### 2.1. Chemicals

6-hydroxy-2,5,7,8-tetramethyl-3,4-dihydrochromene-2-carboxylic acid (trolox), naringenin-7-O-glucoside, ethanol, methanol, formic acid, catechin, vanillin, Folin–Ciocalteu reagent, sodium carbonate, potassium chloride, sodium acetate, acetic acid, hydrochloric acid, 2,2-diphenyl-1-picrylhydrazyl (DPPH), 3-(4,5-Dimethyl-2-thiazolyl)-2,5-diphenyl-2H-tetrazolium bromide (MTT), DMSO, TWEEN20, and LC-MS-grade solvents were purchased from Merck KGaA, Darmstadt, Germany. LC-grade H_2_O (18 MΩ cm) was prepared with a Milli-Q H_2_O purification system (Millipore, Bedford, MA, USA). Malvidin 3-glucoside, peonidin 3-glucoside, delphinidin 3-glucoside, and cyanidin 3-glucoside were obtained from Extrasynthese (Genay CEDEX, France). Four enocianina powders from the skins of grapevine commercially available in Italy and Spain (Aromoss GmbH, Mahlberg-Orschweier, Germany; Dallari Roberto, Bagnolo in Piano, Italy; Secna S.A.U., Chiva, Spain) as food colorant were selected and are here called A, B, C, and D.

### 2.2. Qualitative Analysis by LC-HRMS

A 40 mg/mL stock solution of each enocianina was prepared by dissolving the powder in EtOH/H_2_O (70:30,% *v*/*v*). For the polyphenolic profile analysis, the stock solutions (40 mg/mL) were diluted 1:20 in H_2_O/HCOOH, 100/0.1,% *v*/*v* (mobile phase A) to obtain a final concentration of 2 mg/mL. The internal standard (IS, trolox) was added at a final concentration of 50 µM. Each sample (20 µL) was analyzed in triplicate by LC-HRMS using an LTQ-Orbitrap XL mass spectrometer (Thermo Fisher Scientific, Waltham, MA, USA), as described by Baron et al. [20]. UV-Vis spectrum was also recorded in the scan range of 200–600 nm using a PDA detector (Surveyor, ThermoFinnigan, Milan, Italy). Xcalibur 4.0 and Chromeleon Xpress 6.80 were used for instrument control and spectra analysis. A database was built searching in the literature for the known grapevine phenolic compounds [21,22,23,24,25,26,27,28,29,30,31]. The targeted analysis was performed by searching for all the components listed in the database on the basis of their exact mass (with a mass tolerance of 5 ppm) and the isotopic and fragmentation patterns.

### 2.3. Semiquantitative Analysis of Anthocyanins by LC-MS

Samples were prepared by diluting the stock solutions to a final concentration of 2 mg/mL with mobile phase A (H_2_O/HCOOH 100/2,% *v*/*v*) and adding naringenin-7-O-glucoside (50 µM) as an internal standard. Each sample was analyzed in triplicate by LC-MS, as reported by Baron et al. [32] with brief modifications. Then, 20 µL of sample was injected into the column (Agilent Zorbax SB-C18 reverse-phase column 150 mm, 2.1 mm, particle size 3.5 um, CPS analitica, Milan, Italy) maintained at 50 °C, and the elution was obtained by a multi-step gradient of mobile phase A (H_2_O/HCOOH 100/2,% *v*/*v*) and B (CH_3_CN/CH_3_OH/HCOOH 50/50/2,% *v*/*v*) at a constant flow rate of 250 µL/min. The semi-quantitative analysis was performed using a linear ion trap (LTQ, Thermo Fisher Scientific, Waltham, MA, USA). The ratios between the area under the peak of the extracted ion chromatogram of the *m*/*z* of each analyte and the area of the internal standard were calculated for all anthocyanins in all four samples by averaging the three ratios obtained for each analyte (technical triplicate). The mean ratios for the anthocyanins in each sample were then added together, expressing the results as the relative percentage of each anthocyanin to the total as Equation (1).

(1) $$\mathrm{Relative}\;\mathrm{content}\left(\%\right)=\frac{\mathrm{Analyte}\;\mathrm{area}/\mathrm{IS}\;\mathrm{area}}{\Sigma \left(\mathrm{Analyte}\;\mathrm{area}/\mathrm{IS}\;\mathrm{area}\right)}\times 100$$

### 2.4. Absolute Quantitative Analysis of Anthocyanins by LC-UV

Samples prepared as in Section 2.3 were also analyzed by LC-UV for absolute quantitation of in-house available anthocyanins standards. The chromatographic separation was the same of the semiquantitative analysis, while the detector was a PDA (Surveyor, ThermoFinnigan, Milan, Italy) set to acquire in the range of 200–600 nm. For the absolute quantification, four calibration curves were built using the anthocyanin standards in the following ranges: malvidin 3-glucoside, 5–50 µg/mL; cyanidin 3-glucoside, 0.25–5 µg/mL; peonidin 3-glucoside, 0.5–5 µg; and delphinidin 3-glucoside, 0.25–5 µg/mL.

### 2.5. Total Anthocyanins Content

Total anthocyanin content was determined by UV-Vis spectroscopy according to the method described by Giusti et al. [33]. Total anthocyanin quantification was performed in triplicate for the extracted samples of the four enocianina powders. Stock solutions (40 mg/mL) were diluted with pH 1 buffer (0.025 M potassium chloride solution, titrated to pH 1 with 12 M concentrated HCl) and pH 4.5 buffer (0.4 M sodium acetate solution titrated to pH 4.5 with 12 M concentrated HCl) to obtain a final concentration of 0.5 mg/mL. Each sample was analyzed in technical triplicate. The absorbance of each sample was measured at 520 nm (λ of maximum absorption) and 700 nm using a Shimadzu UV 1900 spectrophotometer (Shimadzu, Milan, Italy). The concentration of anthocyanins in the stock solution was calculated as mg/L as expressed by Equation (2):

(2) $$\mathrm{Concentration}\;\mathrm{of}\;\mathrm{anthocyanins}\;(\mathrm{mg}/L)=\frac{\left(A\times MW\times DF\times 1000\right)}{\left(\varepsilon \times 1\right)}$$

where *A* = (*A*_520_ − *A*_700_)_pH1_ − (*A*_520_ − *A*_700_)_pH4.5_, *MW* = molecular weight of cyanidin glucoside (449.4 g/mol), *DF* = dilution factor, and *ε* = molar absorbance of cyanidin glucoside (26,900 L mol^−1^ cm ^−1^). Results were expressed as reported by Equation (3) as mg of anthocyanins in 100 mg of enocianina powder.

(3) $$\mathrm{Anthocyanins}\;(\mathrm{mg}/100\;\mathrm{mg})=\frac{anthocyanins\;\left(\mathrm{mg}\right)\;in\;the\;stock\;\left(40\;\mathrm{mg}/\mathrm{mL}\right)\times 100}{extracted\;sample\;initial\;weight\;\left(\mathrm{mg}\right)\;}$$

### 2.6. Total Tannins Content

The total tannin content was determined by a colorimetric method using vanillin and HCl as reagents [34]. The assay was performed on a 96-well plate in triplicate. The concentration of 5 mg/mL was chosen for all the extracts, prepared in a H_2_O:EtOH mixture (50:50,% *v*/*v*), so that the absorbance values were within the linearity range of the calibration curve, obtained using catechin (10–1000 µg/mL) as a standard. In each well, the following aliquots were mixed: 150 µL of 4% vanillin (0.8 g vanillin in 20 mL of methanol), 2.5 µL of sample, and 75 µL of 12 M HCl. After shaking for 15 s, a lag phase of 15 min was set before proceeding with the absorbance reading at 500 nm using a PowerWave reader (BioTek’s PowerWave HT, Winooski, VT, USA). Results are expressed as a percentage (%); that is, mg of tannins present in 100 mg of extract.

### 2.7. Total Polyphenols Content

The total polyphenol content was determined by the Folin–Ciocalteu colorimetric method, as reported by Baron et al. [35]; the calibration curve was built using catechin as a standard in a 1–1000 µg/mL range.

### 2.8. DPPH Assay

The antioxidant activity was evaluated with the DPPH assay. Stock solutions of the four enocianinas (40 mg/mL) were diluted with H_2_O:EtOH 50:50 (% *v*/*v*) to obtain concentrations in the range 1–100 µg/mL. An aliquot of 500 µL each solution was mixed with 1 mL of acetate buffer (100 mM, pH 5.5) and 1 mL of ethanol. Finally, 500 µL of an ethanolic solution of DPPH (500 µM) was added and samples were maintained in the dark for 90 min. The absorption was read at 515 nm using a Shimadzu UV 1900 spectrophotometer (Shimadzu, Milan, Italy). The percentage of inhibition was calculated as expressed by Equation (4) and the results are expressed as mean ± SD.

(4) $$I\%=\frac{Abs\;\left(blank\;sample\right)-Abs\;\left(sample\right)}{Abs\;\left(blank\;sample\right)}\times 100$$

### 2.9. NRF2

The four enocianina extracts were evaluated for their ability to modulate the antioxidant response pathway by monitoring the luciferase activity, strictly correlated with NRF2 activation. Experiments were performed using NRF2/ARE Responsive Luciferase Reporter HEK293 stable cell line (Signosis, Santa Clara, CA, USA) in Dulbecco’s modified Eagle medium (DMEM; Lonza, Verviers, Belgium) supplemented with 10% fetal bovine serum (FBS; Gibco, Gaithersburg, MD, USA), 1% Penicillin/Streptomycin (Lonza), and 50 µg/mL of G418 sulfate solution (Promega Corporation, Madison, WI, USA). HEK293 cells were treated with different concentrations (100, 150, 200, and 250 µg/mL) of extracts for 18 h after seeding in a white 96-well plate (BRANDplates^®^, cell grade) at 10,000 cells/well. Subsequently, to avoid any interference on the reading of luciferase activity, media were removed and 100 µL/well of PBS was added. ONE-Glo™ Luciferase Assay Substrate (purchased from Promega Corporation, Madison, WI, USA) (100 µL/well) was directly added to the wells, followed by a luciferase measurement performed using a luminometer (Wallac Victor2 1420, Perkin-Elmer™ Life Science, Monza, Italy). Experiments were performed with biological and technical replicates and results are shown as mean ± SD compared to untreated control cells. Statistical analysis was performed using one-way ANOVA with Bonferroni’s multiple comparisons test (*p* < 0.05 was considered significant). The cell viability was assessed with MTT assay on HEK293 cells treated with all the concentrations of the four enocianinas.

### 2.10. Anti-Inflammatory Activity

The in vitro anti-inflammatory activity of the four extracts was evaluated using a cell model previously described [20]. Briefly, R3/1-Nf-κB cells were seeded (5000 cells/well) in a white 96-well plate (BRANDplates^®^, cell grade). Cells were pre-treated with different concentrations of the extracts (1–250 µg/mL) for 18 h in complete medium (DMEM 10% FBS, 1% L-glutamine, 1% Penicillin/Streptomycin). This process was followed by a 6 h stimulation with 10 ng/mL TNFα. To avoid components’ interference with the reading of the luciferase assay, cells were washed once with 100 µL of warm PBS and 100 µL of DMEM was then added. Subsequently, 100 µL ONE-Glo™ Luciferase Assay Substrate (purchased from Promega Corporation, Madison, WI, USA) was directly added to the wells, followed by a luciferase measurement performed using a luminometer (Wallac Victor2 1420, Perkin-Elmer™ Life Science, Monza, Italy). Experiments were performed with biological and technical replicates and the results are shown as mean ± SD compared to untreated control cells. Statistical analysis was performed using one-way ANOVA with Bonferroni’s multiple comparisons test (*p* < 0.05 was considered significant). The cell viability for all the concentrations tested in the anti-inflammatory assay was verified by MTT assay on R3/1-Nf-κB cells.

### 2.11. MTT Assay

The cell viability for the all the concentrations of enocianinas tested was verified by MTT assay on HEK293 and R3/1-Nf-κB cells in transparent 96-well plates seeded with 10,000 and 4000 cells/well, respectively. After 18 h incubation with enocianinas at the appropriate concentrations (100–250 μg/mL for HEK293 cells and 1–250 μg/mL for R3/1-NF-κB), media were removed, and for the R3/1-NF-κB cell line, one wash with 100 μL PBS occurred. Subsequently, 100 μL/well media not supplemented with FBS and Penicillin/Streptomycin were added, followed by 11 μL MTT reagent (5 mg/mL). After 4 h incubation, media were removed, cells were treated with lysis buffer (100 μL/well) (HCl 8 mM, 5% TWEEN20, DMSO), and the 96-well plate was shaken for 15 min in a plate shaker in the dark. Absorbance at 575 nm and 630 nm was measured using a plate reader (BioTek’s PowerWave HT, Winooski, VT, USA). Cells incubated with DMSO (<0.1%) were used as a control for 100% cell proliferation, while cells incubated with DMSO (3%) were used as a negative control.

### 2.12. Statistical Analysis

The one-way analysis of variance (ANOVA) followed by multiple comparison testing was used to determine any statistically significant differences among the four enocianinas for Nrf2 and Nf-κB responses. Multiple variables analyses of DPPH, total polyphenols, total anthocyanins, and tannins were carried out by building a correlation matrix and computing Pearson correlation calculations. Statistical analysis was performed using GraphPad Prism 9.0.1 software (San Diego, CA, USA, www.graphpad.com, accessed on 7 February 2022). 

## 3. Results and Discussion

### 3.1. Qualitative Profile of Enocianina Extracts Determined by Targeted LC-HRMS Analysis

Qualitative profiling was carried out by LC-HRMS analysis in negative and positive ion modes and using a targeted method. The two polarities were used since anthocyanins are highly ionizable in positive mode, as are phenolic acids in negative ion mode. LC-UV profiles are reported in Supplementary Figures S1 and S2.

**Figure 1:**
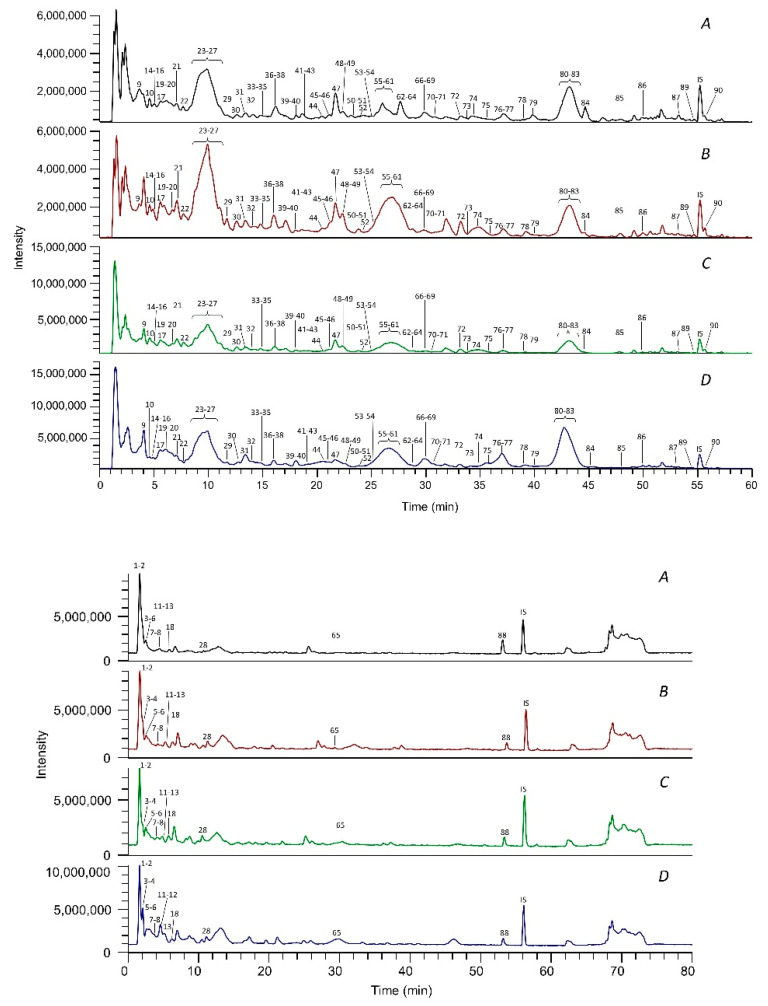
TIC chromatograms of the four commercial enocianinas recorded in positive (**top**) and negative (**bottom**) ion modes. Identified peaks are numbered progressively, according to the elution order, and the assignment of each peak is reported in Supplementary Table S1.

Figure 1 shows the chromatograms of the four enocianinas reported as total ion current (TIC) recorded in positive and negative ion modes. Identified peaks are numbered progressively, according to the elution order. The peak of the internal standard (trolox) is indicated by “IS”. The TIC traces in positive ion mode were almost superimposable among the four samples, and a total of 75 peaks were identified in all four enocianinas samples, thus demonstrating that their qualitative profiles overlap.

The TIC recorded in negative ion mode identified 38 peak ions for all four enocianinas, and also in this case, the qualitative traces relative to the four extracts overlap. The compounds identified only in positive ion mode are 52 and only in negative ion mode are 15, while those identified by both ion modalities are 23. A total of 90 compounds were identified by LC-HRMS, including 41 different anthocyanins, 9 phenolic acids, 1 stilbenoid, 31 flavonols, and 8 flavanols (Supplementary Table S1).

**Figure 2:**
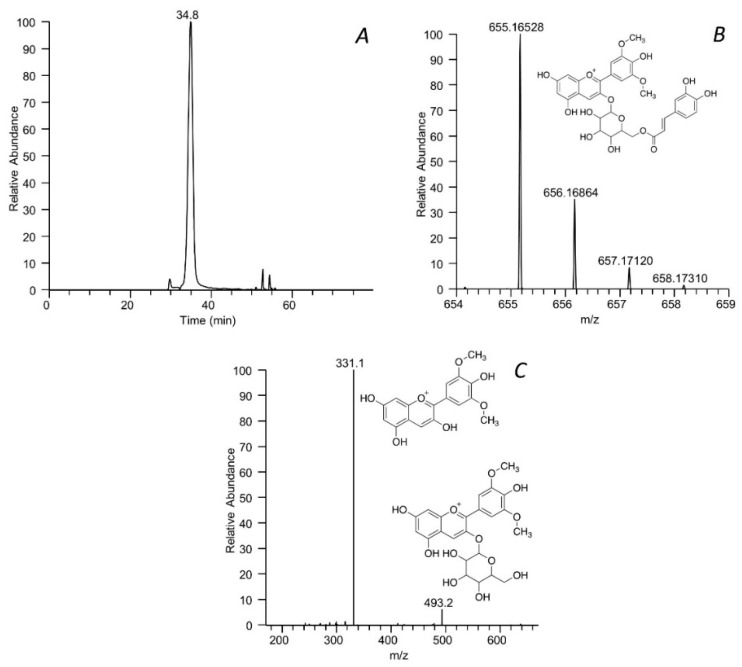
LC-ESI-MS/MS identification of malvidin 3-O-(6″-caffeoyl)-glucoside. Chromatographic peak at 34.8 min (**A**), isotopic (**B**), and fragmentation (**C**) pattern.

The identity of each ion peak was determined by a targeted method. The molecular formula was firstly calculated on the basis of the accurate monoisotopic mass and considering the nitrogen rule, and then searched in an in-house database compiled of analytes so far identified by HRMS in grapevine samples. The compound was then confirmed or identified, in the case of two or more isobaric entries, by matching the experimental and simulated isotopic patterns and taking into account the MS/MS fragmentation. HMDB database and CFM-ID online software were used to assign fragment ions. HMDB contains experimental and predicted MS/MS spectra of around 200,000 metabolites, while CFM-ID can predict the fragmentation spectrum of a given structure, using the mass fragmentation rules, which is then compared to the experimental spectrum. As an example, the identification of malvidin 3-(6″-caffeoyl)-glucoside is described. Figure 2 shows the MS spectrum recorded in positive ion mode of the peak eluting at 34.8 min and characterized by a monoisotopic mass at *m*/*z* 655.16528. By using the tool of Xcalibur, the molecular formula C_32_H_31_O_15_^+^ was calculated and confirmed by comparing the simulated and experimental isotopic patterns. The final attribution was then obtained by matching the MS/MS fragments at *m*/*z* 331 and 493 with those reported in the HMDB database for malvidin 3-O-(6″-caffeoyl)-glucoside. 

Several isomers were identified in both positive and negative ion modes and their identification was made according to their retention time and, in particular, their order of elution, which was compared to that reported by our previous studies or by others, using similar chromatographic conditions. An example is given by differing O-glucosides from O-galactosides, where the former has been reported to elute after the latter by using reverse-phase chromatography [36]. Hence, it was possible to attribute the chromatographic peak at 20.9 min to quercetin 3-O-galactoside and the peak at 22.4 min to quercetin 3-O-glucoside. Another example is given by the structural isomers gallocatechin and epigallogatechin, both characterized by the molecular negative ion at *m*/*z* 305 and MS/MS fragments at *m*/*z* at 125, 179, 219, and 261. On the basis of the order of elution reported in the literature [36], the chromatographic peak at 2.5 min was attributed to gallocatechin and that at 3.8 min to epigallocatechin.

For other isomeric metabolites, identification based on the retention time was not possible as in the case of catechin gallate and epicatechin gallate, laricitrin 3-O-glucoside and laricitrin 3-O-galactoside, and for this reason, both identities are reported.

By using this approach, all the peaks identified in TIC and recorded in positive and negative ion modes were assigned so that the untargeted approach was not required to identify unknown peaks.

Supplementary Table S1 shows the list of identified compounds, ranked based on the elution order.

### 3.2. Quantitative Analyses

The absolute quantitative analysis of available anthocyanins carried out on the LC-UV was obtained with the following calibration curves: malvidin 3-glucoside, *y = 456714* *×* *x − 498813*; peonidin 3-glucoside, *y = 682761* *×* *x − 230734*; delphinidin 3-glucoside, *y = 427567* *×* *x − 1435;* and cyanidin 3-glucoside, *y = 482826* *×* *x + 62097*. The results are expressed as mg of anthocyanins in 100 mg of enocianina (Table 1).

The total content of anthocyanins, tannins, and total polyphenols was determined by spectrophotometry, as reported in the Section 2. The results are reported in Table 2. Anthocyanin content ranged in the four samples from 1.28 to 3.24 mg/100 mg, a concentration range which agrees with that found by other research on enocianina and other by-products of red grapes [4,37]. Tannins were found in a narrower range, from 1.92 to 2.55 mg/100 mg. Polyphenol content in the four extracts ranged from 13 to 23 mg/100 mg and were found in a concentration order not related to that of anthocyanins. Taken together, quantitative and qualitative analyses indicate that enocianina is an extract rich in phenols and polyphenols (90 constituents contained up to 23% *w*/*w*), of which anthocyanins represent a significant but not the most representative flavonoid class (41 constituents, up to 3.24% *w*/*w*). The results are in line with those reported by Prodanov et al. [4], who found that catechins and total condensed tannins represent the main polyphenolic fraction, reaching content up to 10.6% and 15.9%, respectively, while the content of anthocyanin was not higher than 2.65%.

### 3.3. Semi-Quantitative Analysis

**Figure 3:**
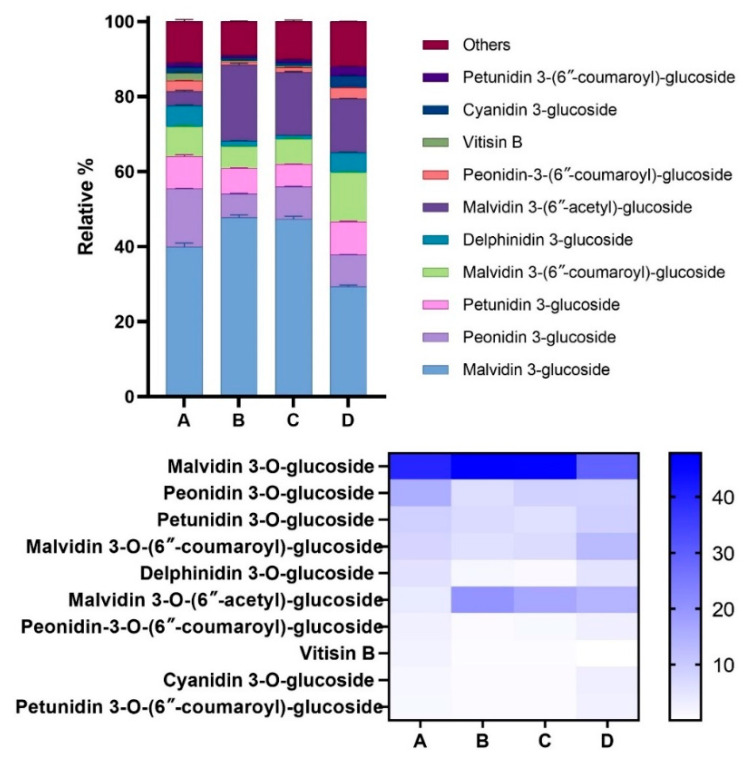
Relative abundances of the anthocyanidins identified in the four commercial enocianinas. Upper panel: Relative abundances are displayed as bars relative to the 10 most abundant components, which account for around 90% of total anthocyanins. Lower panel: Relative abundance of the 10 most abundant components displayed as a heat map.

The relative content of the 41 identified anthocyanins were then calculated for each enocianina sample. Figure 3 (upper and lower panels) shows a graphical representation of the relative amount of the 10 most abundant anthocyanins, accounting for almost 90% of total anthocyanins, while all the values as mean relative percentage ± SD are reported in Table 3. For all four enocianinas, malvidin 3-O-glucoside represents the most abundant component, whose relative content is above 39% for A, B, and C and almost 30% for D. Other abundant anthocyanins contain malvidin as aglycone, and in particular, malvidin 3-O-(6″-acetyl)-glucoside (abundance from 3.86% to 20.24%) and malvidin 3-O-(6″-coumaroyl)-glucoside (abundance from 5.86% to 13.12%). Two other abundant anthocyanins are peonidin 3-O-glucoside (from 6.29% to 15.56%) and petunidin 3-O-glucoside (from 6.78% to 8.90%). 

**Figure 4:**
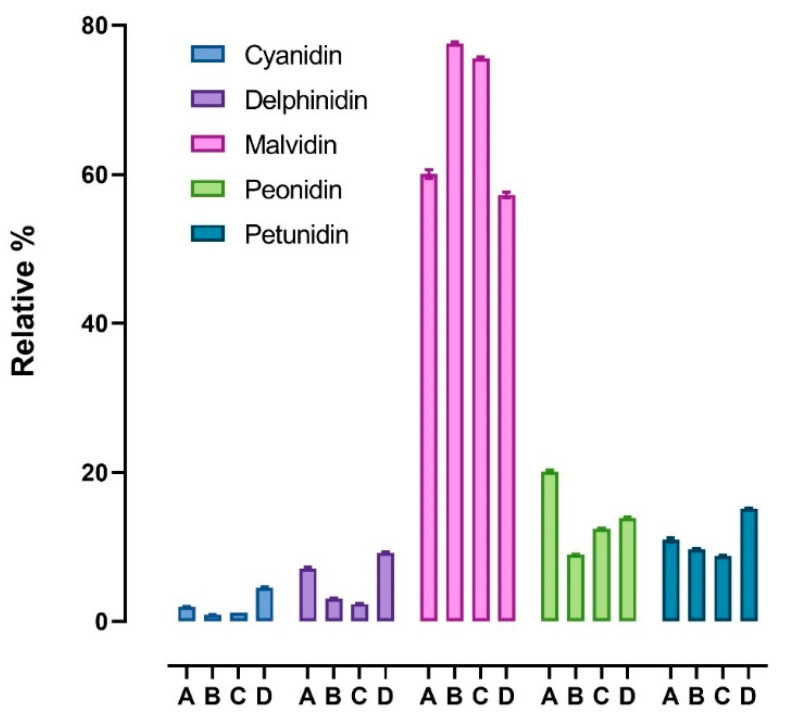
Relative percentages of anthocyanin aglycones (cyanidin, delphinidin, malvidin, peonidin, and petunidin) found in the four samples (A, B, C, D). Data are reported as mean ± SD.

Figure 4 shows the abundance of the five detected aglycons. Malvidin is the most abundant aglycon, followed by peonidin, petunidin, delphinidin, and cyanidin. The results are similar to those reported by Spagna and Pifferi [14], who reported that malvidin is the main aglycon (48%) in enocianina while cyanidin and delphinidin are the least abundant (8% and 4%, respectively). This relative distribution is clearly in accordance with the anthocyanin profiles found in red wine and in general in red grapes and derivatives (red skin grapes), which have a characteristic anthocyanin profile differing with respect to other fruits and vegetables rich in anthocyanins. For instance, cyanidin, which is a reddish-purple (magenta) pigment, is the major pigment in berries [4] and red-colored vegetables such as red sweet potato and purple corn; delphinidin appears as a blue-reddish or purple pigment, which gives the typical blue hue of flowers [2]. 

Two pyranoanthocyanins resulting from the addition of pyruvic acid or acetaldehyde to anthocyanins and which represent important color and functional compounds of red wine were also detected, vitisin A and B. A set of anthocyanin-flavanol pigments was also identified.

**Figure 5:**
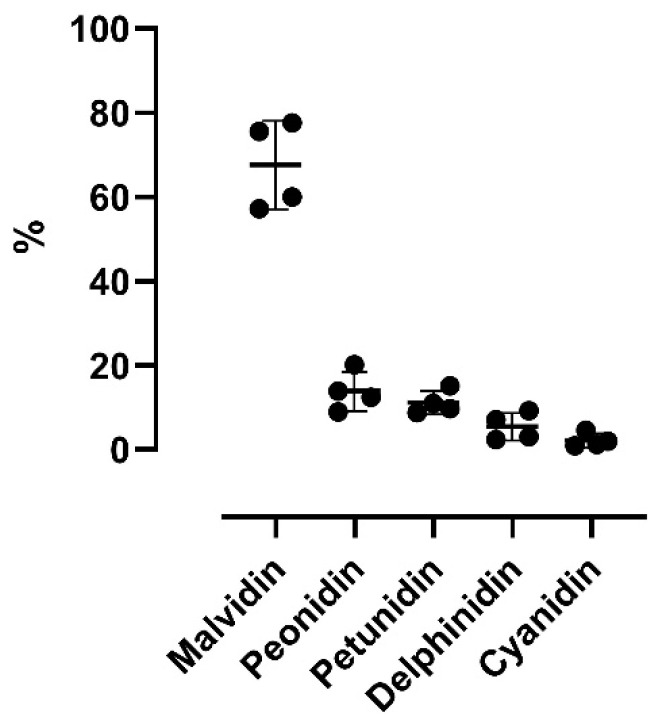
Variability of aglycone content in the four enocianinas. For each aglycone, individual values are reported together with mean and SD.

Figure 5 shows the variability of the content of each aglycone among the four enocianinas. For each aglycone, individual values as determined in each enocianina sample are shown together with mean and SD. The highest variation was found for cyanidin (CV% = 76.8%), followed by delphinidin (60.0%), peonidin (33.8%), petundin (25.1%), and malvidin (15.4%).

**Figure 6:**
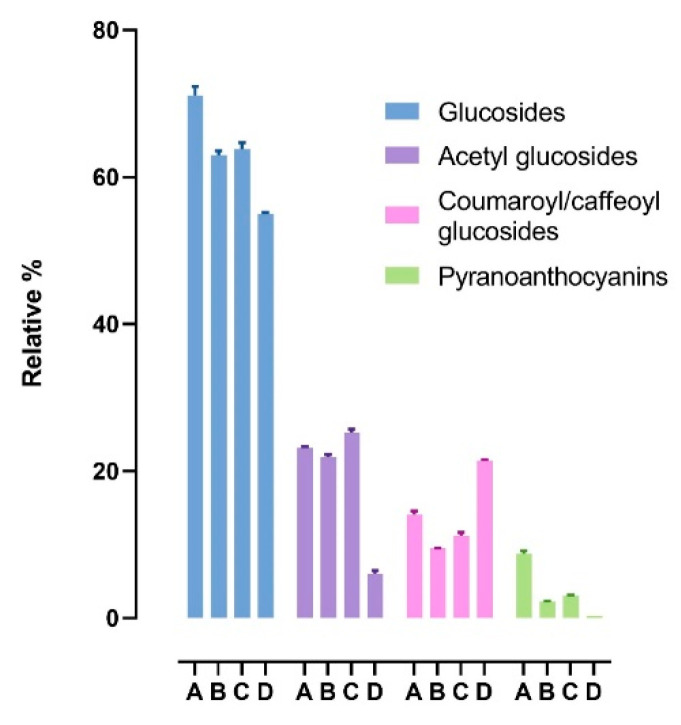
Relative percentages of anthocyanin derivatives (glucosides, acetyl glucosides, coumaroyl/caffeoyl glucosides, pyranoanthocyanins) found in the four samples (A, B, C, D). Data are reported as mean ± SD.

Figure 6 reports the relative content of the moieties attached to the different aglycones (glucosides, acetyl glucosides, coumaroyl/caffeoyl glucosides, and pyranoanthocyanins): glucosides are the most abundant in all four samples, followed by acetyl derivatives, coumaroyl/caffeoyl derivatives (except for sample A, where coumaroyl/caffeoyl derivatives are higher than acetyl derivatives), and pyranoanthocyanins. This trend also reflects the typical widespread profile of *Vitis vinifera* anthocyanins, in which malvidin anthocyanins are primarily non-acetylated derivatives, along with the minority presence of acetyl, coumaroyl, and caffeoyl derivatives. 

**Figure 7:**
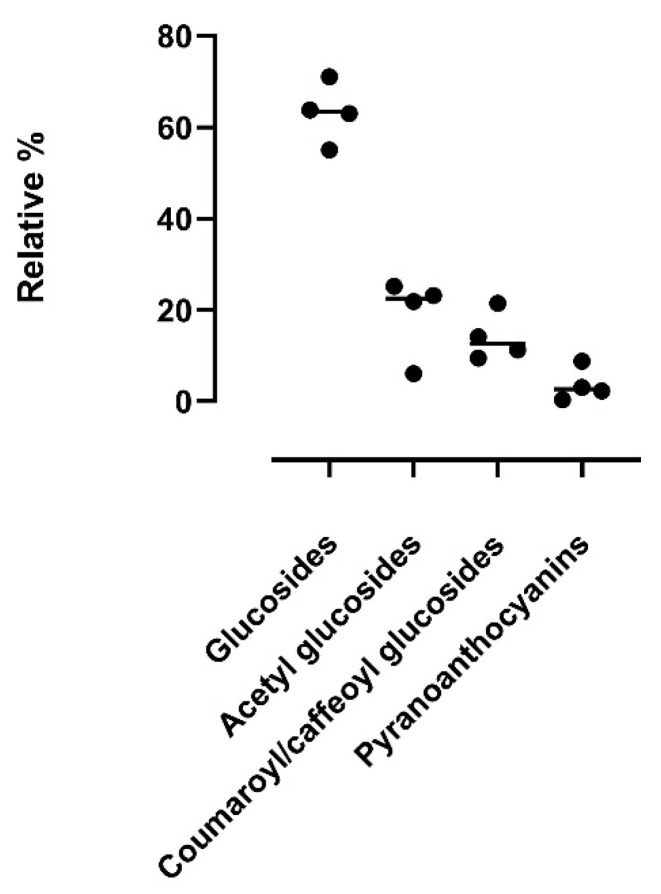
Variability of anthocyanin derivatives found in the four samples. For each sample, individual values are reported together with mean and SD.

Figure 7 reports the variability of the moieties among the four commercial samples. The CV% of glucosides, acetyl-glucosides, coumaroyl/caffeoyl glucosides, and pyranoanthocyanins is 10.38%, 46.09%, 37.43%, and 100.6%, respectively.

### 3.4. Radical Scavenging, Antioxidant and Anti-Inflammatory Activities

**Figure 8:**
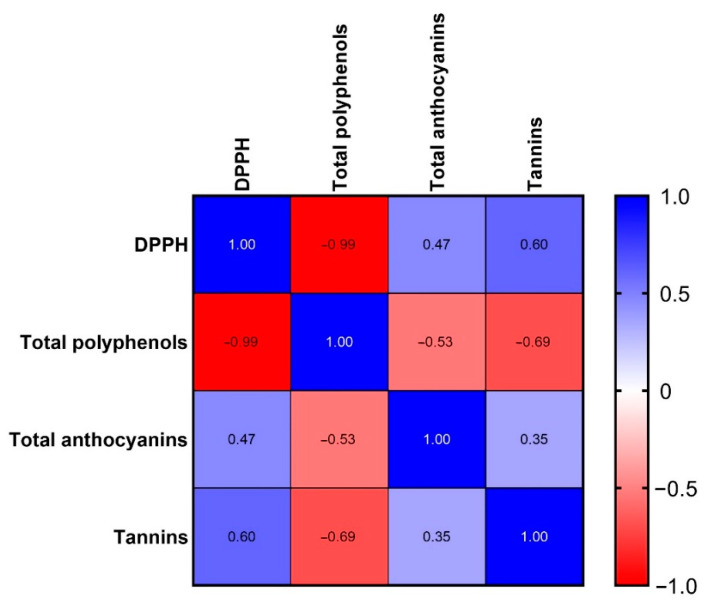
Correlation matrix with Pearson correlation coefficients calculated for DPPH, total polyphenols, total anthocyanins, and tannins. Red and blue colors indicate a negative and positive correlation, respectively. The color intensity indicates the strength of the correlation, as reported in the bar depicted on the right.

Table 4 reports the radical scavenging activity of the four enocianina. Enocianinas A and C show an almost superimposable radical scavenging activity, significantly higher than those of enocianinas B and D. Hence, the order of potency is as follows: A ≈ C > B ≈ D. The lower activity of B and D is related to their total polyphenol content, significantly lower to that found in A and C. The relationships between DPPH, anthocyanins, and polyphenols were evaluated by a correlation matrix and calculating the Pearson correlation coefficients for every data set. As shown in Figure 8, a direct relationship was found between DPPH and polyphenols (Pearson r = −0.991) but not between DPPH and anthocyanins. Hence, based on these data, it seems that the radical scavenging activity of enocianina is primarily mediated by the total polyphenol content rather than that of anthocyanins. This relationship can be firstly explained by the high relative content of total polyphenols with respect to anthocyanins but also to the different structure–activity relationship of flavonoids as radical scavenging compounds.

**Figure 9:**
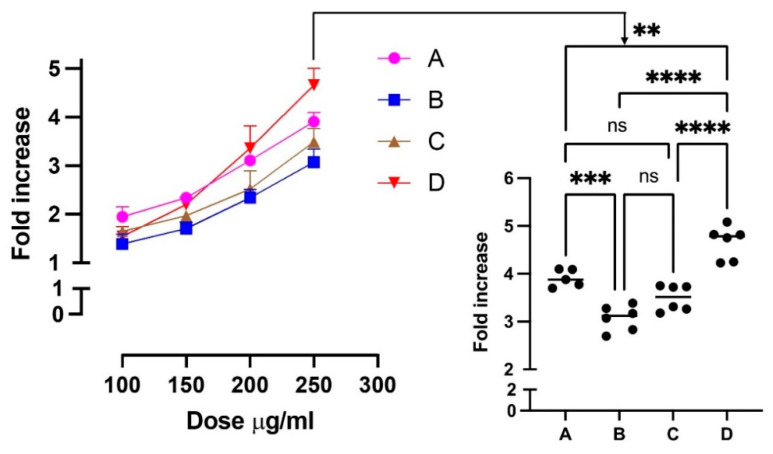
Nrf2 activation by enocianinas. Values are reported as fold increase with respect to control cells. The left panel shows the dose-dependent activity of the four enocianinas in a concentration range between 100 and 250 µg/mL. The right panel shows the one-way ANOVA analysis followed by multi-comparison test for the data relative to the 250 µg/mL concentration. Significances observed for the 250 µg/mL were also observed for the dose of 200 μg/mL (** *p* < 0.01, *** *p* < 0.005, **** *p* < 0.0001).

It is now well accepted that the antioxidant activity of polyphenols, besides a direct radical scavenging mechanism for some radicals, is mediated by an upregulation of the antioxidant enzymes which occurs through the activation of Nrf2, a transcription factor associated with antioxidant enzymes and which plays a master role in redox homeostasis in the cells. Hence, we tested the ability of the four extracts to activate the Nrf2 in a cell model so as to induce an indirect antioxidant effect. All four extracts were found to dose-dependently activate Nrf2 and the order of potency was found as shown in Figure 9, enocianinas D and A being the most effective. The order of potency is opposite to the polyphenol content and to the radical scavenging activity. Activation of Nrf2 by polyphenol compounds is mainly mediated by those compounds bearing an ortho-diphenol moiety which is oxidized to the corresponding quinone, which, being an electrophilic compound, reacts with the thiols of KEAP1, thus releasing Nrf2, which then translocates into the nucleus. Phenols or methoxy derivatives can also be Nrf2 activators, but in this case, a metabolic activation is required to form an ortho or para di-phenol moiety such as the insertion of a hydroxyl group [38], as occurs for resveratrol by the cytochrome P450 enzyme CYP1B1 [39], or a CYP mediate O-demethylation, as reported for sylibin [40]. Such metabolic reactions usually occur in the liver tissue and hence are unlikely to occur in the cells used in the in vitro assay. Hence, we presume that the order of Nrf2 activation is explained by different catechol content in the four extracts. To test this hypothesis, the relative content of each identified compound containing a catechol moiety was determined with respect to the IS and the results are summarized in Table 5, which also reports the catechol index, calculated by summing the relative contents of all the detected catechol compounds. According to the Nrf2 activity, enocianina D was found to be the extract richest in catechol compounds, followed by A, C, and B. Hence, the Nrf2 activation potency is not directly related to the total polyphenols content but to the relative amount of compounds containing a catechol moiety which is responsible for the Nrf2 activation by KEAP1 covalent binding. 

**Figure 10:**
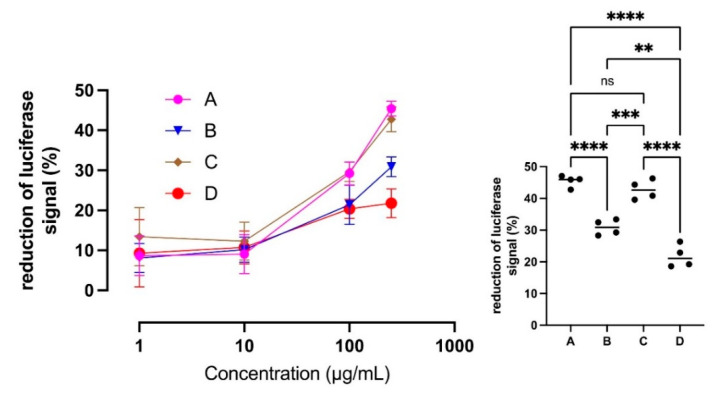
Anti-inflammatory activities of enocianinas. Values are reported as percentage of luciferase signal with respect to cells treated with TNF-α. The left panel shows the dose-dependent activity of the four enocianina in a concentration range between 100 and 250 μg/mL. The right panel shows the one-way ANOVA analysis followed by multi-comparison test for the data relative to the 250 μg/mL concentration (** *p* < 0.01, *** *p* < 0.005, **** *p* < 0.0001).

We then tested the ability of the extracts to inhibit inflammation induced by TNFα in a cell model. All four enocianinas displayed significant and dose-dependent anti-inflammatory activity, as highlighted in Figure 10. The activity was found to be quite overlapping for all the tested compounds at the lowest concentrations, but a significant difference was observed at the highest concentration tested, as reported in Figure 10, right panel, for the dose of 250 μg/mL: enocianinas A and C were almost overlapping (as also shown by of the IC_20_ values in Table 4) and more effective than B (IC_20_ = 115.3 µg/mL), while D was the least potent (IC_20_ = 186.9 µg/mL).

Polyphenols are well reported in the literature for being antioxidant and anti-inflammatory agents. Many examples show their activity in different in vitro models. In particular, Kim et al. studied the antioxidant properties of a specific polyphenol isolated from Anhua dark tea [41], 2S,3R-6-methoxycarbonylgallocatechin (MCGE), in the same model of HEK293 cells we described in the paper. Indeed, dark tea is known for its abundancy of secondary metabolites such as flavonoids and catechins. Interestingly, MCGE demonstrated a potent activity already at a concentration of 0.5 µM with a significant increase in Nrf2 activation. The data presented here agree with what we stated in our work regarding the different antioxidant activity of the four enocianinas. In fact, MCGE presents a 3-hydroxy substituted aromatic ring which probably allows the oxidation to quinone, essential to react with thiols of KEAP1 and to release Nrf2, which then translocates into the nucleus. Regarding the anti-inflammatory activity, the same model reported in this work was used to test the activity of two different extracts by our research group: a red grape skin extract and two bergamot extracts [20,35]. In these papers, it is also highlighted how the content of polyphenols was strictly correlated with the anti-inflammatory activity, which is in line with what we found regarding the four enocianinas.

The results indicate that the anti-inflammatory activity of enocianina can be only in part attributed to an Nrf2 activation mechanism and hence to the catechol-containing compounds since the order of potency between Nrf2 activation, catechol index, and anti-inflammatory activity does not match. The anti-inflammatory activity was found to be in line with the polyphenol content, and higher content of polyphenols in enocianina corresponds to higher radical scavenging and anti-inflammatory activity. Hence, the anti-inflammatory activity of enocianina is the result of the activity of polyphenols, which act through different mechanisms, including radical scavenging, Nrf2 activation, and others not yet clarified. For instance, the anti-inflammatory effect of malvidin, the main component of enocianina, has been related to gene expression modulation (reducing the expression of proinflammatory genes), not dependent on an Nrf2 activation mechanism [42].

## 4. Conclusions

By using an LC-HRMS approach, we performed a detailed qualitative and semi-quantitative profiling of enocianina, with the results indicating that enocianina is an extract rich in polyphenols of which anthocyanins represent a significant, but not the most representative, flavonoid class. Enocianina was found to exert direct (radical scavenging effect) and indirect (Nrf2 activation) antioxidant activity, the latter related to the catechol constituents. Finally, all the tested enocianinas showed an anti-inflammatory activity, which was found to be strictly related to the polyphenol content and which could be explained not only by the radical scavenging and Nrf2 activation but also by other mechanisms related to non-catechol polyphenols.

In conclusion, enocianina is a mixture of highly valuable phytochemicals which exert antioxidant and anti-inflammatory effects, involving both catechol and non-catechol derivatives.

## Figures and Tables

**Table 1:** Absolute quantitative content of anthocyanin determined by LC-UV.

	Malvidin 3-Glucoside	Peonidin 3-Glucoside	Delphinidin 3-Glucoside	Cyanidin 3-Glucoside
Code	Mean ± SD	Mean ± SD	Mean ± SD	Mean ± SD
	mg/100 mg	mg/100 mg	mg/100 mg	mg/100 mg
**A**	1.056 ± 0.027	0.247 ± 0.049	0.293 ± 0.013	0.050 ± 0.007
**B**	1.131 ± 0.027	0.083 ± 0.003	0.060 ± 0.003	0.007 ± 0.001
**C**	1.302 ± 0.032	0.190 ± 0.012	0.049 ± 0.001	0.015 ± 0.001
**D**	1.744 ± 0.026	0.363 ± 0.007	0.575 ± 0.012	0.223 ± 0.013

**Table 2:** Total content of anthocyanins, tannins, and total polyphenols as determined by spectrophotometry.

	Anthocyanins	Tannins	Total Polyphenols
Code	Mean ± SD	Mean ± SD	Mean ± SD
	mg/100 mg	mg/100 mg	mg/100 mg
**A**	1.66 ± 0.11	1.92 ± 0.96	23.539 ± 1.438
**B**	1.28 ± 0.02	2.39 ± 0.69	14.640 ± 0.903
**C**	1.46 ± 0.03	2.51 ± 0.23	21.063 ± 2.113
**D**	3.24 ± 0.07	2.55 ± 0.06	13.069 ± 0.706

**Table 3:** Relative percentages (mean ± SD) of the 41 anthocyanins identified.

Name	A	B	C	D
Malvidin 3-glucoside	39.846 ± 1.070	47.799 ± 0.684	47.332 ± 0.751	29.325 ± 0.427
Peonidin 3-glucoside	15.563 ± 0.064	6.292 ± 0.090	8.599 ± 0.113	8.444 ± 0.035
Petunidin 3-glucoside	8.685 ± 0.338	6.787 ± 0.076	5.996 ± 0.060	8.902 ± 0.090
Malvidin 3-(6″-coumaroyl)-glucoside	7.958 ± 0.293	5.861 ± 0.031	6.734 ± 0.227	13.124 ± 0.058
Delphinidin 3-glucoside	5.508 ± 0.217	1.484 ± 0.017	1.047 ± 0.004	5.263 ± 0.145
Malvidin 3-(6″-acetyl)-glucoside	3.862 ± 0.246	20.243 ± 0.412	16.779 ± 0.194	14.354 ± 0.077
Peonidin-3-(6″-coumaroyl)-glucoside	2.789 ± 0.069	0.786 ± 0.016	1.236 ± 0.041	2.962 ± 0.052
Vitisin B	2.104 ± 0.090	0.407 ± 0.006	0.477 ± 0.013	0.029 ± 0.002
Cyanidin 3-glucoside	1.481 ± 0.066	0.660 ± 0.014	0.845 ± 0.014	3.095 ± 0.057
Petunidin 3-(6″-coumaroyl)-glucoside	1.172 ± 0.040	0.715 ± 0.014	0.830 ± 0.031	2.509 ± 0.022
Vitisin A	1.124 ± 0.035	0.446 ± 0.006	0.920 ± 0.016	0.067 ± 0.001
Delphinidin 3-(6″-p-coumaroyl)-glucoside	1.054 ± 0.043	0.441 ± 0.006	0.487 ± 0.021	1.861 ± 0.017
Peonidin 3-(6″-acetyl)-glucoside	1.041 ± 0.092	1.748 ± 0.042	2.413 ± 0.078	2.471 ± 0.038
Malvidin 3-O-glucoside-8-ethyl-(epi)catechin isomer 3	0.933 ± 0.048	0.160 ± 0.007	0.071 ± 0.007	0.008 ± 0.001
Malvidin 3-(6″-caffeoyl)-glucoside	0.644 ± 0.032	1.471 ± 0.013	1.652 ± 0.077	0.149 ± 0.003
Malvidin 3-O-(6′′-p coumaroyl)glucoside acetaldehyde	0.643 ± 0.016	0.064 ± 0.002	0.068 ± 0.006	0.009 ± 0.000
Petunidin 3-(6″-acetyl)-glucoside	0.640 ± 0.046	2.002 ± 0.049	1.731 ± 0.058	3.667 ± 0.047
Malvidin 3-O-glucoside-8-ethyl-(epi)catechin isomer 2	0.603 ± 0.025	0.095 ± 0.002	0.049 ± 0.005	0.006 ± 0.000
Malvidin 3-O-(6′′-p coumaroyl)glucoside ethyl-catechin	0.572 ± 0.035	0.068 ± 0.003	0.044 ± 0.001	0.005 ± 0.003
Delphinidin 3-(6″-acetyl)-glucoside	0.384 ± 0.018	1.073 ± 0.034	0.768 ± 0.037	2.053 ± 0.003
Cyanidin 3-O-(6′′-p-coumaroyl)glucoside	0.361 ± 0.023	0.100 ± 0.003	0.164 ± 0.008	0.796 ± 0.019
Peonidin 3-O-glucoside-pyruvate	0.334 ± 0.010	0.067 ± 0.002	0.167 ± 0.002	0.012 ± 0.000
Malvidin 3-O-glucoside-8-ethyl-(epi)catechin isomer 4	0.317 ± 0.034	0.065 ± 0.003	0.031 ± 0.005	0.003 ± 0.001
Malvidin 3-O-(6′′-p coumaroyl)glucoside-pyruvate	0.285 ± 0.018	0.066 ± 0.000	0.127 ± 0.002	0.031 ± 0.001
Petunidin 3-O-glucoside-acetaldehyde	0.278 ± 0.014	0.068 ± 0.005	0.095 ± 0.005	0.023 ± 0.001
Malvidin 3-O-(6′′-acetyl)glucoside-acetaldehyde	0.254 ± 0.021	0.080 ± 0.003	0.031 ± 0.000	0.010 ± 0.000
Peonidin 3-O-glucoside-8-ethyl-(epi)catechin isomer 2	0.247 ± 0.016	0.018 ± 0.001	0.012 ± 0.001	0.001 ± 0.000
Malvidin 3-O-glucoside-4-vinyl-(epi)catechin	0.230 ± 0.016	0.153 ± 0.002	0.306 ± 0.019	0.011 ± 0.001
Malvidin 3-O-glucoside-8-ethyl-(epi)catechin isomer 1	0.159 ± 0.014	0.031 ± 0.002	0.020 ± 0.002	0.003 ± 0.000
Peonidin 3-O-glucoside-8-ethyl-(epi)catechin isomer 1	0.156 ± 0.010	0.009 ± 0.001	0.005 ± 0.001	0.001 ± 0.000
Cyanidin-3-acetylglucoside	0.122 ± 0.013	0.146 ± 0.003	0.192 ± 0.010	0.656 ± 0.011
Malvidin 3-O-(6′′-acetyl)glucoside-pyruvate	0.122 ± 0.005	0.135 ± 0.005	0.093 ± 0.001	0.040 ± 0.001
Petunidin 3-(6″-caffeoyl)-glucoside	0.111 ± 0.009	0.118 ± 0.005	0.133 ± 0.007	0.028 ± 0.001
Malvidin 3-O-glucoside-acetone	0.076 ± 0.009	0.147 ± 0.016	0.348 ± 0.026	0.010 ± 0.001
Delphinidin 3-O-glucoside-8-ethyl-(epi)catechin	0.074 ± 0.003	0.040 ± 0.002	0.029 ± 0.002	0.014 ± 0.001
Malvidin 3-O-glucosidepyruvate procyanidin dimer	0.055 ± 0.006	0.048 ± 0.003	0.075 ± 0.004	0.001 ± 0.001
Malvidin 3-O-glucoside-acetaldehyde	0.048 ± 0.005	0.044 ± 0.005	0.053 ± 0.008	0.011 ± 0.000
Malvidin 3-O-glucoside-pyruvate	0.044 ± 0.011	0.015 ± 0.002	0.012 ± 0.001	0.009 ± 0.001
Petunidin 3-O-(6′′-p-coumaroyl)glucoside-8-ethyl-(epi)catechin	0.040 ± 0.002	0.006 ± 0.001	0.003 ± 0.001	0.002 ± 0.000
Malvidin 3-O-(6′′-p-coumaroyl)glucoside-4-vinylphenol	0.035 ± 0.002	0.007 ± 0.001	0.005 ± 0.001	0.020 ± 0.001
Malvidin 3-O-glucosidepyruvate procyanidin dimer	0.028 ± 0.004	0.020 ± 0.001	0.010 ± 0.001	0.002 ± 0.000

**Table 4:** Antioxidant and anti-inflammatory activities of enocianina.

	Radical Scavenging Activity	Anti-Inflammatory Activity
Code	IC_50_ µg/mL	IC_20_ µg/mL
	(Mean ± SD)	(Mean ± SD)
**A**	9.582 ± 0.871	68.4 ± 14.3
**B**	16.093 ± 2.173	115.3 ± 25.1
**C**	10.552 ± 1.371	52.2 ± 16.9
**D**	16.389 ± 3.472	186.9 ± 48.2

**Table 5:** Relative amount calculated with respect to the IS of each compound in enocianina containing the catechol moiety and catechol index as a sum of the relative amount of each catechol compound.

Catechols	A	B	C	D
Delphinidin 3-glucoside	5.66	1.65	0.85	10.15
Procyandin B peak1	0.71	2.47	2.75	2.40
Cyanidin 3-glucoside	1.29	0.69	0.71	6.84
Procyanidin trimer peak 1	0.16	0.96	0.86	0.65
Catechin	0.41	1.29	1.30	0.99
Procyanidin trimer peak 2	0.20	0.77	0.76	0.59
Petunidin 3-glucoside	9.23	7.12	6.54	21.52
Procyanidin B peak4	0.82	2.27	2.58	2.20
Epicatechin	0.45	1.08	1.38	0.83
Procyanidin trimer peak 3	0.33	1.12	1.17	0.97
Petunidin 3-O-glucoside-acetaldehyde	0.27	0.06	0.06	0.02
Procyanidin tetramer	0.09	0.40	0.42	0.20
Delphinidin 3-O-glucoside-8-ethyl-(epi)catechin	0.31	0.03	0.02	0.02
Delphinidin 3-(6″-acetyl)-glucoside	0.70	2.28	1.46	10.36
Myricetin 3-glucuronide	0.35	0.47	0.39	0.28
Myricetin 3-glucoside	0.86	1.65	1.76	0.83
Myricetin dihexoside	0.03	0.48	0.27	0.01
Cyanidin-3-acetylglucoside	0.28	0.38	0.45	3.04
Catechin gallate/epicatechin 3-gallate	0.31	0.48	0.60	0.03
Petunidin 3-(6″-acetyl)-glucoside	1.36	5.39	3.97	23.98
Quercetin 3-galactoside	0.52	0.61	0.66	0.35
Quercetin 3-glucuronide	8.03	7.13	8.81	4.61
Quercetin 3-glucoside	1.90	1.09	1.90	0.09
Dihydroquercetin-3-rhamnoside	0.56	0.49	0.83	1.96
Malvidin 3-O-glucoside-8-ethyl-(epi)catechin isomer 1	0.76	0.24	0.14	0.02
Laricitrin-3-glucoside/Laricitrin 3-galactoside	0.59	1.83	2.19	0.87
Peonidin 3-O-glucoside-8-ethyl-(epi)catechin isomer 1	0.75	0.09	0.06	0.01
Malvidin 3-O-glucoside-8-ethyl-(epi)catechin isomer 2	2.68	0.48	0.23	0.05
Peonidin 3-O-glucoside-8-ethyl-(epi)catechin isomer 2	0.99	0.08	0.05	0.01
Malvidin 3-O-glucosidepyruvate procyanidin dimer 1	0.18	0.10	0.15	0.00
Malvidin 3-O-glucoside-8-ethyl-(epi)catechin isomer 3	4.21	1.00	0.46	0.10
Petunidin 3-(6″-caffeoyl)-glucoside	0.22	0.39	0.44	0.21
Malvidin 3-O-glucosidepyruvate procyanidin dimer 2	0.22	0.26	0.32	0.02
Malvidin 3-O-glucoside-8-ethyl-(epi)catechin isomer 4	13.42	0.63	0.30	0.04
Quercetin-3-rhamnoside	0.55	0.48	0.75	2.21
Delphinidin 3-(6″-coumaroyl)-glucoside	2.75	2.10	2.08	14.88
Myricetin	0.21	0.24	0.22	0.85
Malvidin 3-(6″-caffeoyl)-glucoside	3.12	9.84	9.87	1.73
Cyanidin 3-O-(6′′-p-coumaroyl)glucoside	1.59	0.58	0.98	7.40
Petunidin 3-O-(6′′-p-coumaroyl)glucoside-8-ethyl-(epi)catechin	0.42	0.11	0.07	0.01
Petunidin 3-(6″-coumaroyl)-glucoside	5.51	5.09	5.83	23.98
Malvidin 3-O-glucoside-4-vinyl-(epi)catechin	0.92	0.95	1.30	0.02
Malvidin 3-O-(6′′-p coumaroyl)glucoside ethyl-catechin	6.12	0.96	0.79	0.11
Quercetin	2.42	2.70	3.76	0.22
Laricitrin	0.05	0.09	0.08	0.10
**Catechol index**	**82.49**	**68.59**	**70.54**	**145.73**

## Data Availability

Data is contained within the article and supplementary material.

## Supplementary Materials

The following supporting information can be downloaded at: https://www.mdpi.com/article/10.3390/antiox11061187/s1, Figure S1: LC-UV profile of the four enocianina acquired in the range 200-600 nm; Figure S2: Magnification of Figure S2; Table S1: Compounds identified by LC-ESI-MS in positive and negative ion modes are ordered on the basis of the retention time. Compounds shown in the TIC recorded in negative ion mode (lower panel of Figure 1) are in italic.
