# Supplementary material for: Liquid Chromatography–High-Resolution Mass Spectrometry (LC-HRMS) Profiling of Commercial Enocianina and Evaluation of Their Antioxidant and Anti-Inflammatory Activity
Source: Antioxidants (Basel). 2022 Jun 16;11(6):1187. doi: 10.3390/antiox11061187 (PMC9231191; doi:10.3390/antiox11061187)
Supplement: Supplementary file 1 [file antioxidants-11-01187-s001.zip › antioxidants-1722440-supplementary.pdf]

Table S1. Compounds identified by LC-ESI-MS in positive and negative ion modes are ordered on the basis of the retention time. Compounds shown in the TIC recorded in negative ion mode (lower panel of Figure 1) are in italic.

Peak	Compound name	RT	$[M-H]^-_{exp}$	Δ ppm	MS/MS	$[M+H]^+_{exp}$	Δ ppm	MS/MS
1	Gallic acid	1.9	169.01400	1.775	123 - 125 - 169			
2	Galloyl glucose	2.1	331.06653	0.030	169 - 191 - 241 - 271			
3	Hexoside of protocatechuic acid	2.4	315.07181	0.666	153			
4	Gallocatechin	2.5	305.06628	0.524	125 - 179 - 219 - 261			
5	Protocatechuic acid	3.2	153.01910	2.091	109 - 123 - 153			
6	Caftaric acid	3.5	311.03958	-0.675	149 - 179			
7	Epigallocatechin	3.8	305.06628	0.524	125 - 179 - 219 - 261			
8	Caffeoyl glucoside 1	3.9	341.0878	1.612	179			
9	Delphinidin 3-O-glucoside	4				465.10240	1.914	303
10	Procyandin B peak1	4.1	577.13452	-0.121	289-407-425	579.14910	1.951	291 - 409 - 427
11	Procyanidin B peak2	4.4	577.13452	-0.121	289-407			
12	Caffeoyl glucoside 2	4.6	341.0878	1.612	179			
13	Coutaric acid	5.4	295.04544	0.169	149 - 163			
14	Cyanidin 3-O-glucoside	5.4				449.10773	1.447	287
15	Procyanidin trimer peak 1	5.4				867.21252	1.268	577-579
16	Catechin	5.6	289.07144	0.795	125 - 245	291.08661	0.824	123 - 139 - 147 - 151 - 165
17	Procyanidin trimer peak 2	5.9				867.21332	0.345	577-579
18	Procyanidin B peak3	6.1	577.13434	-0.433	289-407-425			
19	Petunidin 3-O-glucoside	6.4				479.11800	1.962	317
20	*p*-coumaroyl-glucose 1	6.7	325.09241	0.215	163	327.10751	1.436	309
21	Procyanidin B peak4	7.1	577.13434	-0.433	289-407-425	579.14940	1.433	291 - 409 - 427
22	Fertaric acid	7.4	325.05646	1.568	193	327.07114	1.375	133 - 196 - 177 - 309
23	*p*-coumaroyl-glucose 2	8.3	325.09241	0.215	163 - 235 - 265	327.10757	1.253	147 - 309
24	Epicatechin	8.7	289.07159	1.314	109 - 125 - 245	291.08643	1.442	123 - 139 - 151 - 165 - 273
25	Peonidin 3-O-glucoside	9				463.12320	1.792	301
26	Malvidin 3-O-glucoside	9.8				493.13360	2.008	331
27	Procyanidin trimer peak 3	11				867.21210	1.752	577-579
28	Ethyl gallate	11.7	197.04530	1.522	125 - 151 - 153 - 169			
29	Petunidin 3-O-glucoside-acetaldehyde	11.9				503.11771	1.371	341
30	Procyanidin tetramer	13				1155.2761	0.770	-
31	Delphinidin 3-O-glucoside-8-ethyl-(epi)catechin	13.2				781.19702	1.228	329-491-619

32	Delphinidin 3-O-(6″-acetyl)-glucoside	14				507.11290	1.893	303
33	Myricetin 3-glucuronide	14.2	493.0618	-0.060	317	495.07650	1.959	319
34	Peonidin 3-O-glucoside-pyruvate	14.2				531.11279	0.997	369
35	Myricetin 3-O-glucoside	14.8	479.08295	0.814	317	481.09730	1.871	319
36	Myricetin dihexoside	16.1				643.14996	1.648	319 - 481
37	Vitisin A	16.2				561.12330	1.996	399
38	Malvidin 3-O-glucoside-pyruvate	16.3				561.12317	1.265	399
39	Piceid	18.2	389.1239	0.668	227	391.13861	1.712	229
40	Vitisin B	18.5				517.13370	1.713	355
41	Malvidin 3-O-glucoside-acetaldehyde	18.9				517.13348	1.102	355
42	Cyanidin 3-O-acetylglucoside	19.1				491.11832	1.262	287
43	Catechin gallate/epicatechin gallate	19.2	441.08234	0.385	289	443.09705	1.715	153 - 273 - 291
44	Malvidin 3-O-(6″-acetyl)-glucoside-pyruvate	20.6				603.13403	0.696	399
45	Petunidin 3-O-(6″-acetyl)-glucoside	20.7				521.12848	1.976	317
46	Quercetin 3-O-galactoside	20.9	463.08786	0.453	301	465.10245	1.806	303
47	Quercetin 3-O-glucuronide	21.6	477.06769	1.634	301	479.08176	1.649	303
48	Quercetin 3-O-glucoside	22.4	463.08795	0.647	301	465.10242	1.871	303
49	Dihydroquercetin 3-O-rhamnoside	22.6				451.12317	1.884	305 - 415 - 433
50	Malvidin 3-O-glucoside-8-ethyl-(epi)catechin isomer 1	23.7				809.22827	0.580	357-519-647
51	Laricitrin 3-O-glucoside / Laricitrin 3-O-galactoside	23.8	493.0986	0.790	331	495.1146	-1.514	333
52	Peonidin 3-O-glucoside-8-ethyl-(epi)catechin isomer 1	24.2				779.2179	1.039	327-489-617
53	Malvidin 3-O-glucoside-acetone	24.9				531.14923	0.884	369
54	Malvidin 3-O-(6″-acetyl)-glucoside-acetaldehyde	25.1				559.1441	0.929	355
55	Malvidin 3-O-glucoside-8-ethyl-(epi)catechin isomer 2	25.9				809.22852	0.271	357-519-647
56	Peonidin 3-O-(6″-acetyl)-glucoside	26				505.13361	1.940	301
57	Peonidin 3-O-glucoside-8-ethyl-(epi)catechin isomer 2	26.6				779.21777	1.206	327-489-617
58	Kaempferol 3-O-galactoside	26.6	447.09286	0.290	285	449.10761	1.692	287
59	Malvidin 3-O-(6″-acetyl)-glucoside	27.1				535.14429	1.607	331
60	Malvidin 3-O-glucosidepyruvate procyanidin dimer 1	27.2				1093.2596	1.573	641-803-943
61	Malvidin 3-O-glucoside-8-ethyl-(epi)catechin isomer 3	27.7				809.22809	0.803	357-519-647
62	Petunidin 3-O-(6″-caffeoyl)-glucoside	28.5				641.14966	1.512	302 - 317 - 479
63	Malvidin 3-O-glucosidepyruvate procyanidin dimer 2	28.9				1093.2603	1.015	641-803-943
64	Kaempferol 3-O-glucuronide	29.4	461.07202	0.043	285	463.08673	1.965	287
65	Isorhamnetin 3-O-glucoside	29.7	477.10336	0.125	299 - 315			
66	Kaempferol 3-O-glucoside	29.9	447.09286	0.290	285	449.10751	1.914	287

67	Malvidin 3-O-glucoside-8-ethyl-(epi)catechin isomer 4	29.9				809.22821	0.654	357-519-647
68	Quercetin 3-O-rhamnoside	30				449.10773	1.425	287 - 303
69	Delphinidin 3-O-(6″-coumaroyl)-glucoside	30.3				611.13892	1.882	303
70	Dihydrokaempferol 3-O-rhamnoside	30.8				435.12869	0.965	289 - 322 - 399 - 417
71	Myricetin	31.2				319.04498	1.253	153 - 165 - 245 - 273 - 301
72	Syringetin 3-O-glucoside	33.1	507.11444	1.143	344	509.12869	1.591	347
73	Malvidin 3-O-(6″-coumaroyl)-glucoside-pyruvate	34.2				707.16022	1.385	399
74	Malvidin 3-O-(6″-caffeoyl)-glucoside	34.8				655.16528	1.526	331 - 493
75	Cyanidin 3-O-(6″-coumaroyl)-glucoside	35.8				595.1438	1.377	287
76	Petunidin 3-O-(6″-coumaroyl-)glucoside-8-ethyl-(epi)catechin	36.5				941.24957	0.318	343-651
77	Petunidin 3-O-(6″-coumaroyl)-glucoside	36.6				625.15448	1.983	317
78	Kaempferol 3-O-rhamnoside	38.9				433.11270	1.755	287
79	Malvidin 3-O-(6″-coumaroyl)-glucoside acetaldehyde	39.9				663.17023	1.719	355
80	Peonidin 3-O-(6″-coumaroyl)-glucoside	42.3				609.15967	1.855	301
81	Malvidin 3-O-glucoside-4-vinyl-(epi)catechin	42.4				805.19708	0.447	491-643
82	Malvidin 3-O-(6″-coumaroyl)-glucoside	43.2				639.17023	1.783	331
83	Malvidin 3-O-(6″-coumaroyl)-glucoside ethyl-catechin	44.7				955.2652	0.889	357-665
84	Syringetin 3-O-(6''-acetyl)-glucoside	45.2	549.12463	0.382	344	551.1391	1.741	347
85	Quercetin	47.8	301.03531	1.594	151 - 165 - 179 - 301	303.0499	1.880	137 - 153 - 229 - 247 - 285
86	Laricitrin	49.9				333.06061	1.261	-
87	Malvidin 3-O-(6″-coumaroyl)-glucoside-4-vinylphenol	53.3				755.19666	0.516	447
88	Syringetin	53.9	345.06131	0.782	315			
89	Kaempferol	54.7	285.04007	0.561	173 - 229 - 241 - 285	287.05508	1.637	121 - 153 - 213 - 241
90	Isorhamnetin	55.6	315.05069	0.666	151 - 315	317.06558	1.703	285-302

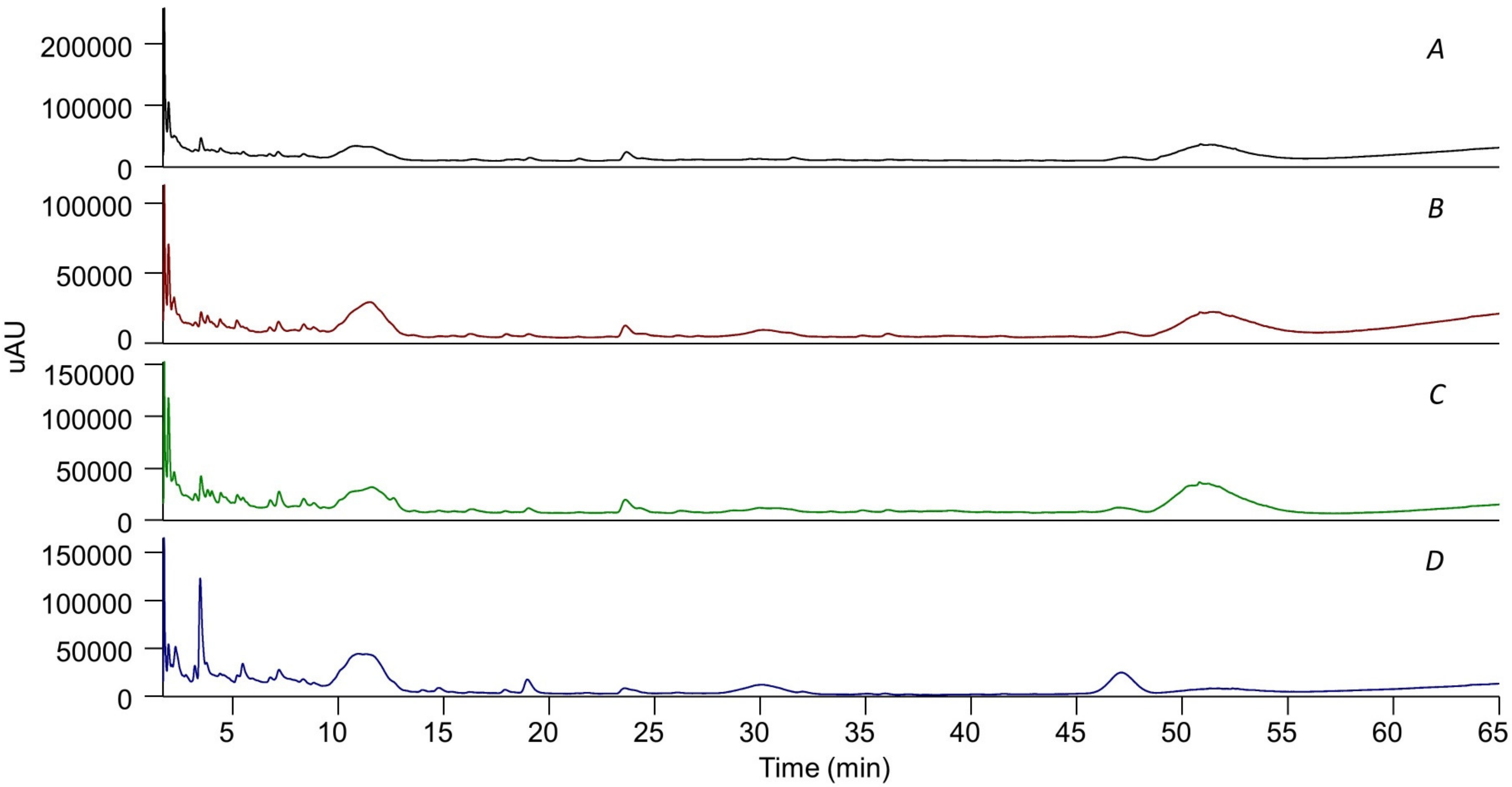

Figure S1. LC-UV profile of the four enocianina acquired in the range 200-600 nm.

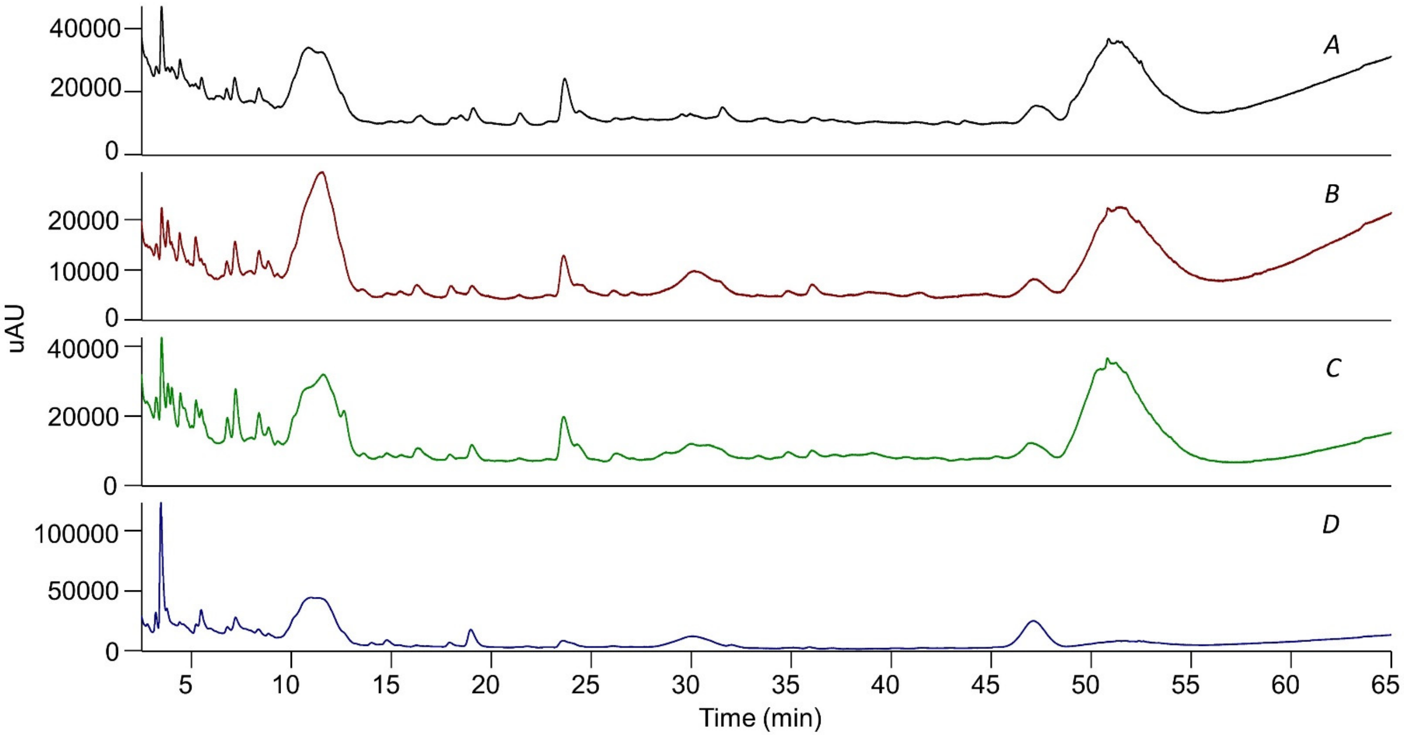

Figure S2. Magnification of Figure S2.
